# Multiple global change stressor effects on phytoplankton nutrient acquisition in a future ocean

**DOI:** 10.1098/rstb.2019.0706

**Published:** 2020-03-23

**Authors:** Dedmer B. Van de Waal, Elena Litchman

**Affiliations:** 1 Department of Aquatic Ecology, Netherlands Institute of Ecology (NIOO-KNAW), Droevendaalsesteeg 10, Wageningen 6871 CM, The Netherlands; 2 W. K. Kellogg Biological Station, Michigan State University, 3700 E. Gull Lake Drive, Hickory Corners, MI 49060, USA; 3 Department of Integrative Biology, Michigan State University, 288 Farm Lane, East Lansing, MI 48824, USA

**Keywords:** climate change, phytoplankton, traits, resource allocation, eco-physiology

## Abstract

Predicting the effects of multiple global change stressors on microbial communities remains a challenge because of the complex interactions among those factors. Here, we explore the combined effects of major global change stressors on nutrient acquisition traits in marine phytoplankton. Nutrient limitation constrains phytoplankton production in large parts of the present-day oceans, and is expected to increase owing to climate change, potentially favouring small phytoplankton that are better adapted to oligotrophic conditions. However, other stressors, such as elevated *p*CO_2_, rising temperatures and higher light levels, may reduce general metabolic and photosynthetic costs, allowing the reallocation of energy to the acquisition of increasingly limiting nutrients. We propose that this energy reallocation in response to major global change stressors may be more effective in large-celled phytoplankton species and, thus, could indirectly benefit large-more than small-celled phytoplankton, offsetting, at least partially, competitive disadvantages of large cells in a future ocean. Thus, considering the size-dependent responses to multiple stressors may provide a more nuanced understanding of how different microbial groups would fare in the future climate and what effects that would have on ecosystem functioning.

This article is part of the theme issue ‘Conceptual challenges in microbial community ecology’.

## 1. Primary production in a future ocean

Marine phytoplankton play a pivotal role in the oceanic carbon cycle and fuel the marine food web. Consequently, climate-driven shifts in oceanic primary production will have major consequences not only for carbon export, but also for the structure and functioning of the entire marine biome. Understanding how multiple global change stressors act simultaneously affecting phytoplankton productivity and community structure is difficult because of the complex interactions among those factors [1]. Looking at traits that are involved in potential phytoplankton responses to different global change stressors and determining how these traits are affected by those stressors, together with assessing potential trade-offs that may be involved, could help us improve the conceptual understanding of multiple stressor effects on different phytoplankton groups.

Both elevated *p*CO_2_ and warming are major global change stressors impacting marine phytoplankton, and their effects can be direct as well as indirect. For instance, elevated *p*CO_2_ may directly facilitate oceanic primary production through enhanced photosynthesis [2–4]. Yet, the effects are species- and even strain-specific, depending on distinct inorganic carbon acquisition strategies, including the operation and regulation of carbon concentrating mechanisms (CCMs) [5–8]. Various studies, however, have shown that elevated *p*CO_2_ does not necessarily enhance primary production, or may even have negative effects, e.g. caused by concomitant changes in carbonate chemistry such as reduced pH (i.e. ocean acidification; [9,10]). Warming directly affects organisms by enhancing their metabolic rates [11,12]. Specifically, warming may enhance respiration rates more than photosynthesis, and thus possibly lead to declines in net oceanic carbon fixation [13,14].

Besides the direct effects on primary production, warming is also expected to enhance thermal stratification at low and mid latitudes, preventing nutrients from deep waters entering the well-lit surface mixed layer, thus exacerbating phytoplankton nutrient limitation and reducing primary production [15–17]. Moreover, enhanced nutrient trapping in the Southern Ocean due to climatic changes was shown to increase nutrient export to the deep ocean, further strengthening nutrient limitation [18]. Thus, present-day oceanic phytoplankton primary production is already constrained by the availability of key nutrients such as nitrogen, phosphorus and iron [19], and this limitation is expected to increase in a future ocean. Phytoplankton have developed a range of traits to deal with prevailing low-nutrient conditions. These nutrient utilization traits may change in response not only to increased nutrient limitation but to major global change stressors as well, such as higher *p*CO_2_ and temperatures.

How do increased *p*CO_2_, warming and nutrient limitation interact to modify phytoplankton physiology, ecology and ecosystem impacts? No doubt, the effects are complex and varied. To illustrate this complexity, we focus on how phytoplankton nutrient acquisition may be modified by elevated *p*CO_2_, warming and higher light availabilities, and what consequences this may have on oceanic ecosystems. Inspired by trait-based approaches in ecology [20–22], we propose using traits to understand the combined effects of climate change factors and nutrient limitation on marine phytoplankton. Specifically, we highlight the impacts of climate change on nutrient acquisition at the individual level through phenotypic plasticity, at the population level through genotype-specific responses with potential consequences for evolutionary adaptation, and at the community level through climate-driven species sorting, revealing unexpected scenarios for shifts in community size structure.

## 2. Plasticity of nutrient acquisition traits

### (a) Elevated *p*CO_2_ and warming

**Figure 1.**
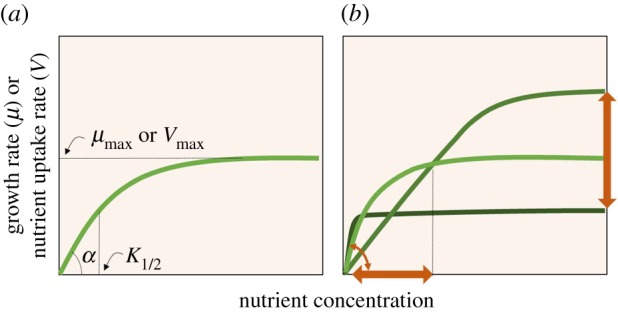
Conceptual overview of nutrient acquisition traits (*a*) and putative impacts of climate change (warming and elevated *p*CO_2_) on these traits in marine phytoplankton (*b*). Nutrient acquisition traits include maximum growth (*µ*_max_) or uptake (*V*_max_) rate, half-saturation concentration (*K*_1/2_) and the nutrient or growth uptake affinity (*α*). Brown arrows in (*b*) indicate potential effects of climatic change (darker shades) on nutrient acquisition traits.

Nutrient acquisition in phytoplankton approximates a hyperbolic function, with uptake rates and growth rates steeply increasing at low nutrient concentrations toward saturation when nutrient is in excess [23–26]. Key nutrient acquisition traits include the maximum uptake rate (*V*_max_) or maximum growth rate (*µ*_max_), and the half-saturation constant (*K*_1/2_), which describes the concentration of a nutrient where nutrient uptake or growth equals half of the maximum rate. Nutrient uptake or growth affinity (*α*) combines both traits, representing the initial slope following *V*_max_/*K*_1/2_ and *µ*_max_/*K*_1/2_, respectively (figure 1*a*) [24,27]. Climate-driven increases in nutrient limitation may thus likely benefit phytoplankton with high nutrient uptake or growth affinities, attained either through plastic responses or through evolutionary selection. In addition to the uptake traits, the minimum nutrient requirement, minimum nutrient quota *Q*_min_, is important in determining nutrient competitive abilities, which can be expressed as scaled uptake affinity $V_{\max}/K_{1/2}Q_{\min}\;$ [28]. In general, smaller-celled species tend to have better competitive abilities [28], so that they would have a competitive advantage in the future more oligotrophic ocean.

**Figure 2.**
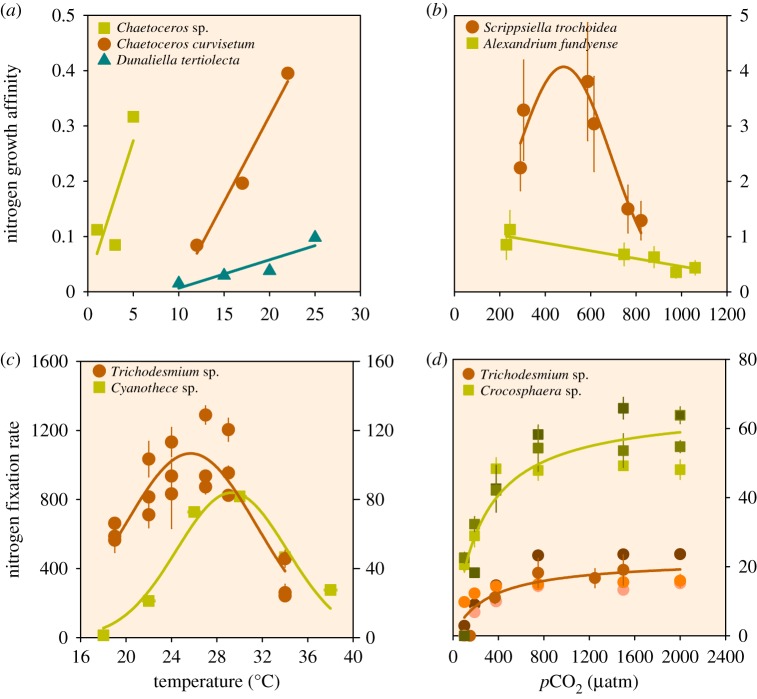
Impacts of climate change factors on nitrogen acquisition. Temperature and CO_2_ effects on (*a*,*b*) nitrogen growth affinity, and (*c*,*d*) nitrogen fixation in marine phytoplankton. (*a*,*b*) Nitrogen growth affinity is defined as the initial slope of the Monod relationship, expressed as l µmol^−1^ day^−1^. Nitrogen fixation is expressed as (*c*) fmol C_2_H_4_ cell^−1^ (12 h)^−1^ for *Cyanothece* (left *y*-axis), and as mmol N_2_ (mol C)^−1^ h^−1^ for *Trichodesmium* (right *y*-axis), and as (*d*) µmol N (mg Chl a)^−1^ h^−1^ for different species or strains (indicated by different colour shades) of *Croocosphaera* and *Trichodesmium*. Figures were redrawn from Reay *et al.* [27] with permission from the American Society for Microbiology (*a*), from Eberlein *et al.* [31] and Breitbarth *et al.* [32] under the Creative Commons Attribution License (*b*,*c*), and from Brauer *et al.* [33] and Hutchins *et al.* [34] (*c*,*d*). If unavailable, data were extracted using Engauge Digitizer [35].

Phytoplankton possess high phenotypic plasticity and can strongly modulate their physiology in response to elevated *p*CO_2_ and temperature. Warming may reduce the energetic and elemental costs for overall metabolism, and with elevated *p*CO_2_ the energetic costs of carbon acquisition could be reduced, notably by down-regulation of energy-demanding CCMs [4,5,29]. Consequently, cells may reallocate energy and/or elements to enhance the uptake of a limiting resource, leading to higher nutrient uptake or growth affinities (figure 1*b*). Indeed, at higher temperatures phytoplankton seem to have higher nitrogen uptake rates (for ammonium and urea, but not nitrate) [30], and higher nutrient growth affinities (figure 2*a*) [27]. Similarly, elevated *p*CO_2_ also led to higher net nitrogen assimilation rates (i.e. nitrogen quota multiplied by growth rate) in two dinoflagellate species. This was accompanied, however, by a disproportional increase in their *K*_1/2_ for nitrogen, highlighting a potential trade-off between the rate at which nitrogen is assimilated and the relative affinity for nitrogen [31]. Consequently, nitrogen growth affinities (i.e. *µ*_max_/*K*_1/2_) were at an optimum or decreased with elevated *p*CO_2_ (figure 2*b*). These findings were mainly explained by a shift toward higher investments in nitrogen-rich functional compounds, such as alkaloid toxins and chlorophyll *a*. Alternatively, the CO_2_-driven down-regulation of CCMs may enhance photo-oxidative stress, leading to increased energetic and elemental costs (e.g. nitrogen) associated to photo-inhibition [36–38], which may, in turn, lead to reduced nitrogen growth affinities.

A major source of (bioavailable) nitrogen in the open ocean is the N_2_ fixed by diazotrophic cyanobacteria [39] and released into the water column. Thus, changes in N_2_ fixation could significantly alter N budgets in oligotrophic oceans. Nitrogen fixation has been shown to be strongly temperature-dependent, with optimum rates in warm, low latitude tropical and subtropical regions (figure 2*c*) [32,33]. Although warming generally enhances N_2_ fixation rates, it may also cause oxygen inhibition of the responsible enzyme nitrogenase, thereby possibly leading to a decline in N_2_ fixation rates at high temperatures [33,40]. Nitrogen fixation rates were shown to generally increase at higher *p*CO_2_, from present-day levels of around 400 ppm to approximately 750 ppm [41–43], beyond which N_2_ fixation rates levelled off (figure 2*d*). These patterns show that N_2_ fixation can be limited by CO_2_, but also that there is a maximum rate at CO_2_ levels above 1000 ppm [34]. Although elevated *p*CO_2_ was shown to be beneficial for N_2_ fixation, a decrease in pH may possibly inhibit it owing to a decrease in nitrogenase efficiency, resulting in declined growth and N_2_ fixation rates [44,45, but see 46].

Besides nitrogen and phosphorus, iron is a major limiting resource for oceanic primary production as well, particularly in the Southern Ocean [19]. Similar to nitrogen and phosphorus, the uptake and assimilation of iron were also shown to be affected by temperature. Specifically, along with enhancing N_2_ fixation, warming increased iron use efficiency in a marine diazotroph (*Trichodesmium*), and this could even offset the effect of iron limitation [47]. At the same time, however, the inhibitory effect of decreasing pH was most apparent under Fe-limiting conditions [44,45]. Whether elevated *p*CO_2_ would promote N_2_ fixation may, thus, depend on the availability of Fe, and further work is needed to elucidate the interactive effects of Fe and CO_2_ on N_2_ fixation in marine diazotrophic cyanobacteria.

### (b) Increased light availabilities

Enhanced thermal stratification of the oceanic waters may lead to shallowing of the upper mixed layer, which may, together with sea ice retreat, enhance the relative light availability in the ocean surface layers and thereby stimulate primary production [48,49]. With higher relative light availabilities, the costs of light capture may be reduced and, thus, could allow reallocation of energy and/or elements towards nutrient acquisition. Indeed, higher light levels were shown to enhance N_2_ fixation rates in diazotrophic cyanobacteria [33,50]. Moreover, cellular chlorophyll *a* content in various phytoplankton species decreased with higher light intensities [51], which may reduce nitrogen demand for synthesizing these pigments [52]. Increasing light availability can also directly decrease nutrient demands of phytoplankton by reducing their elemental quota [53], though these responses may vary among species [54]. Higher light availabilities combined with elevated *p*CO_2_, however, may cause photo-oxidative stress, thereby leading to reduced primary production [37,55, but see 56]. Despite being beneficial to photosynthesis, the impact of enhanced light levels will thus depend on the availability of other resources, and may possibly become detrimental.

## 3. Favouring the small…

Climate-driven depletion of nutrients may shift phytoplankton communities towards dominance by species with low nutrient requirements, high nutrient uptake efficiencies, and a high flexibility to shunt excess energy towards nutrient acquisition. Being small seems a particularly good strategy to deal with nutrient depletion, as (absolute) nutrient requirements are proportional to size [25,57]. Moreover, because of their high surface-to-volume ratio, small cells have higher growth and nutrient uptake affinities, and are less likely to become diffusion-limited [25,58–60]. Consequently, smaller-sized phytoplankton generally dominate phytoplankton biomass in the open ocean where nutrients are depleted and primary production is low, while larger-celled phytoplankton are generally more dominant in more productive coastal waters [61,62].

**Figure 3.**
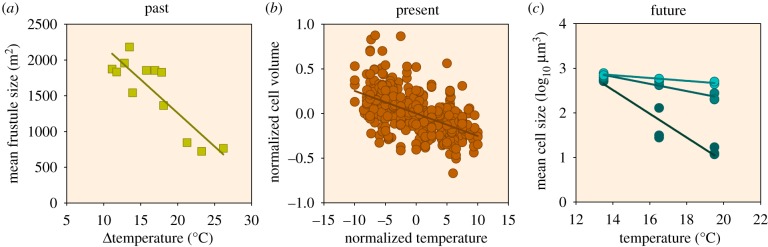
Relationships between cell size and temperature in the past, present and future. Size of diatom frustules from sediment cores as a function of reconstructed past temperatures (*a*), cell volumes of contemporary phytoplankton from culture experiments of brackish water and marine phytoplankton (*b*), and cell sizes of a Baltic Sea phytoplankton community in response to experimental warming combined with nutrient limitation from a high (darker shade) to a low (lighter shade) level of nutrient limitation (*c*). The *y*-axis in (*b*) indicates the difference between cell volume at any temperature and the estimated volume at 15°C, normalized to this mean volume, and the *x*-axis indicates difference between the tested temperature and 15°C (for further details, see Atkinson *et al.* [63]). Figures were redrawn from Finkel *et al.* [64] (copyright © (2005) National Academy of Sciences) (*a*), from Atkinson *et al.* [63] with permission from Royal Society Publishing (*b*), and from Peter & Sommer [65] under the Creative Commons Attribution License (*c*). If unavailable, data were extracted using Engauge Digitizer [35].

With climate-driven declines in nutrient availabilities, phytoplankton communities may thus possibly shift towards small-celled species. Indeed, the size of diatom frustules, indicative of diatom cell size, was shown to be inversely correlated with temperature variations over the past approximately 65 Myr (figure 3*a*). In other words, warmer periods had smaller diatoms dominant, which could have resulted from the reductions in nutrient availability due to enhanced thermal stratification [64]. Also in contemporary marine phytoplankton, cell size usually decreases with temperature (figure 3*b*) [63]. Similarly, experimental warming led to a shift in the phytoplankton community toward smaller phytoplankton species, which was most prominent under high-nutrient stress (figure 3*c*) [65]. This is in line with climate change scenarios tested with a global Earth system model, which projected a shift toward smaller phytoplankton species, particularly at higher latitudes [66].

## 4. …and the large?

Although nutrient depletion generally favours small phytoplankton, climate-driven reallocation of energy and/or elements may be particularly beneficial for large species. First, large species are more diffusion-limited compared with small species and may thus benefit relatively more from enhanced CO_2_ diffusion rates. For example, elevated *p*CO_2_ was shown to favour growth of larger diatoms [67], and to shift phytoplankton communities to larger species [67,68]. Second, large species have relatively high elemental investments in light capturing, because of the lower absorption efficiencies compared with small-celled phytoplankton [69]. Consequently, large species may benefit relatively more from increased light availability caused by shallower mixing layer depths in a warmer ocean, as they can reallocate more resources and energy from light harvesting to nutrient acquisition. Third, large species also tend to be more flexible in size, with a proportionately greater possible reduction compared with smaller species, because smaller-celled species are closer to their minimum structural demands and, therefore, have limited cell size flexibility [70]. Large species may, thus, have a greater ability to reduce cell size and benefit from the associated increases in surface-to-volume ratio. Fourth, some larger phytoplankton taxa possess vacuoles that increase their surface-to-volume ratio, thereby enhancing the effective surface area for nutrient transport [71,72]. Fifth, these vacuoles serve as storage compartments for nutrients, particularly advantageous in fluctuating nutrient conditions [73]. With climate change, storm intensities are predicted to increase [74], which may temporarily enhance nutrient concentrations in the surface waters by mixing with nutrient-rich deeper waters, and was shown, as consequence, to promote primary production and favour large diatoms [75,76]. Lastly, cell size is generally correlated with genome size [77,78], and processes such as adaptive gene loss and genomic streamlining may optimize nutrient acquisition traits in small phytoplankton species, particularly in more stable environments [79]. Conversely, it is conceivable that larger cells may have a greater gene redundancy, leading to more resilient traits [8], which may provide a competitive advantage in dynamic environments. In summary, the higher flexibility of larger phytoplankton species in response to direct and indirect effects of warming and elevated *p*CO_2_ may, at least partially, offset their competitive disadvantage in nutrient acquisition.

## 5. Evolution of nutrient acquisition traits

Impacts of climate change on marine phytoplankton will depend not only on their plastic responses, but also on their potential to adapt evolutionarily through selection on standing genetic variation or novel mutations [80,81]. Adaptation to elevated *p*CO_2_ and warming was observed in various phytoplankton species across major marine phytoplankton groups [82–88]. Yet, evolutionary responses to elevated *p*CO_2_ seem to be diverse, and may, furthermore, differ in direction compared with the observed plastic responses of phenotypes [89]. However, evolutionary changes observed in coccolithophores that adapted to elevated *p*CO_2_ were consistent with their plastic responses and, at least partially, offset fitness losses [82,89,90].

**Figure 4.**
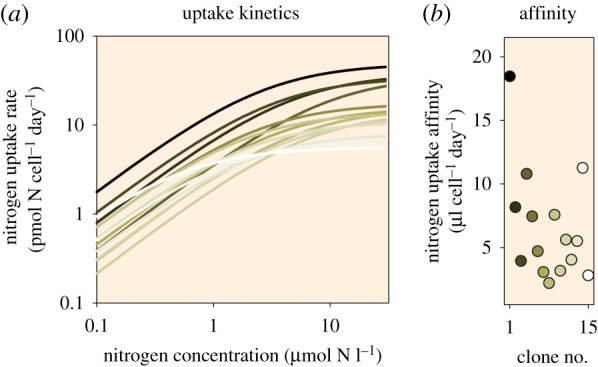
Intraspecific variation in nitrogen uptake kinetics. (*a*) Nitrogen uptake rates as a function of nitrogen concentrations, and (*b*) uptake affinities of various dinoflagellate *Alexandrium ostenfeldii* clones (indicated by different colour shades). Data were modified from Brandenburg *et al.* [95] and are available through Brandenburg *et al.* [96].

Evolutionary adaptation toward elevated *p*CO_2_ was particularly evident under environmental conditions that decreased fitness [89]. It is therefore conceivable that a decline in fitness due to increased nutrient limitation might be compensated by adaptation through increased nutrient uptake affinities. Existing intraspecific genetic and phenotypic diversity of marine phytoplankton populations is substantial and thus provides the basis for adaptation through the selection of best-fit genotypes [91–94]. With regard to nutrient acquisition, populations of the dinoflagellate *Alexandrium ostenfeldii* were shown to exhibit a large intraspecific variation in nutrient uptake kinetics, demonstrating a wide range of nitrogen uptake affinities (figure 4) [95]. This suggests a large potential for selection of clones with higher nutrient uptake affinities when nutrients become (more) limiting.

## 6. Concluding remarks and future directions

We have described how a trait-based ecological approach may help understand the interactive impacts of climate change factors and nutrient limitation on marine phytoplankton, highlighting possible shifts in nutrient acquisition through elevated *p*CO_2_, warming or changes in light availabilities. We hypothesize that climate-driven exacerbation of nutrient limitation may be partially counteracted by the concomitant increases in *p*CO_2_, temperature and relative light availabilities, which may benefit large phytoplankton species capable of reallocating greater resources to nutrient acquisition more than small species and thus, at least partially, offset their competitive disadvantages. This could lead to different outcomes for phytoplankton size distributions, which, in turn, would have different effects on ecosystem processes and food-web dynamics. The next step would be to incorporate energy or resource reallocation in mechanistic models, investigating the magnitude of possible direct and indirect effects of simultaneously acting stressors, and link these to food-web and ecosystem models, thereby generating process-based predictions for oceanic ecosystems.

Obviously, global environmental changes involve a multitude of factors that may affect phytoplankton in diverse ways, maybe differently from what we propose here. By highlighting the complex interplay of several global change stressors on phytoplankton nutrient acquisition, we argue that investigating how multiple stressors may interact to modify phytoplankton traits should be an urgent research priority, requiring collaborations of phytoplankton physiologists, ecologists and modellers. Also, we note that taking into account interacting stressors may yield different predictions compared with when stressors are considered in isolation. For example, recent work showed that nutrient limitation may make phytoplankton more vulnerable to rising temperatures by decreasing their temperature optima and impeding evolutionary adaptation to warming [97,98]. Using a trait-based framework for a better mechanistic understanding of trait flexibility in different phytoplankton size classes under the combined changes in *p*CO_2_, temperature and resource availabilities, as well as other anticipated environmental change stressors, should further improve our predictions of the future oceanic primary production and ecosystem dynamics.

## Data accessibility

This article has no additional data.
